# Myeloperoxidase-Related Chlorination Activity Is Positively Associated with Circulating Ceruloplasmin in Chronic Heart Failure Patients: Relationship with Neurohormonal, Inflammatory, and Nutritional Parameters

**DOI:** 10.1155/2015/691693

**Published:** 2015-10-11

**Authors:** Aderville Cabassi, Simone Maurizio Binno, Stefano Tedeschi, Gallia Graiani, Cinzia Galizia, Michele Bianconcini, Pietro Coghi, Federica Fellini, Livia Ruffini, Paolo Govoni, Massimo Piepoli, Stefano Perlini, Giuseppe Regolisti, Enrico Fiaccadori

**Affiliations:** ^1^Cardiorenal Research Unit, Department of Clinical and Experimental Medicine, University of Parma Medical School, Italy; ^2^Laboratory of Experimental Physiopathology, Department of Clinical and Experimental Medicine, University of Parma Medical School, Italy; ^3^Dentistry School, Department of Clinical and Experimental Medicine, University of Parma Medical School, Italy; ^4^Cardiology Clinic, Azienda Ospedaliera-Universitaria di Parma, Via Gramsci 14, 43126 Parma, Italy; ^5^Nuclear Medicine Unit, Azienda Ospedaliera-Universitaria di Parma, Via Gramsci 14, 43126 Parma, Italy; ^6^Histology and Embryology Unit, Department of Biomedical, Biotechnological and Translational Sciences, University of Parma Medical School, Italy; ^7^Heart Failure Unit, Cardiology Department, Guglielmo da Saliceto Hospital, Piacenza, Italy; ^8^Department of Internal Medicine, University of Pavia, Italy

## Abstract

*Rationale*. Heart failure (HF) is accompanied by the development of an imbalance between oxygen- and nitric oxide-derived free radical production leading to protein nitration. Both chlorinating and peroxidase cycle of Myeloperoxidase (MPO) contribute to oxidative and nitrosative stress and are involved in tyrosine nitration of protein. Ceruloplasmin (Cp) has antioxidant function through its ferroxidase I (FeO_*x*_I) activity and has recently been proposed as a physiological defense mechanism against MPO inappropriate actions. *Objective*. We investigated the relationship between plasma MPO-related chlorinating activity, Cp and FeO_*x*_I, and nitrosative stress, inflammatory, neurohormonal, and nutritional biomarkers in HF patients. *Methods and Results*. In chronic HF patients (*n* = 81, 76 ± 9 years, NYHA Class II (26); Class III (29); Class IV (26)) and age-matched controls (*n* = 17, 75 ± 11 years, CTR), plasma MPO chlorinating activity, Cp, FeO_*x*_I, nitrated protein, free Malondialdehyde, BNP, norepinephrine, hsCRP, albumin, and prealbumin were measured. Plasma MPO chlorinating activity, Cp, BNP, norepinephrine, and hsCRP were increased in HF versus CTR. FeO_*x*_I, albumin, and prealbumin were decreased in HF. MPO-related chlorinating activity was positively related to Cp (*r* = 0.363, *P* < 0.001), nitrated protein, hsCRP, and BNP and inversely to albumin. *Conclusions*. Plasma MPO chlorinated activity is increased in elderly chronic HF patients and positively associated with Cp, inflammatory, neurohormonal, and nitrosative parameters suggesting a role in HF progression.

## 1. Introduction

**Figure 1:**
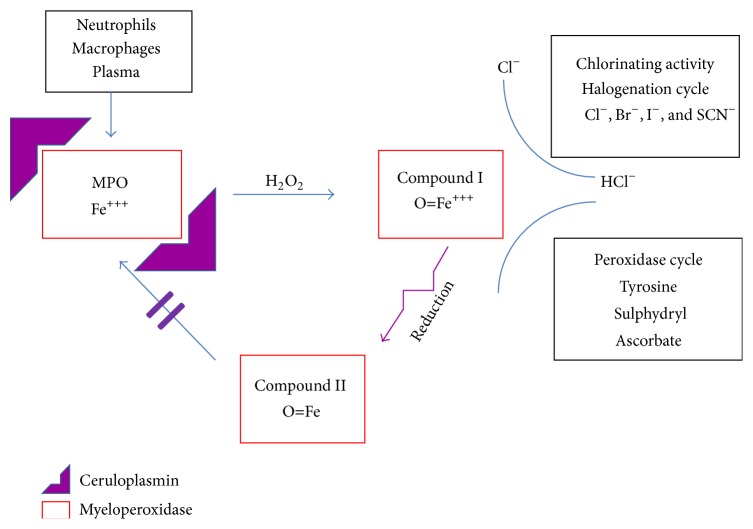
Schematic diagram indicating the relationship between Myeloperoxidase-related chlorinating activity and Ceruloplasmin (Cp). Ceruloplasmin binding to MPO determines reduction of the active Compound I to Compound II and prevents the recycling of Compound II back to the active enzyme.

Heart failure (HF) disease is accompanied by the development of an imbalance between oxygen- and nitric oxide-derived free radical production and the ability of the protective shield represented by a series of antioxidant enzymes to scavenge and buffer the overwhelming quantity of radical species generated [1]. Myeloperoxidase (MPO) is a glycosylated heme-enzyme, mainly stored in the primary azurophilic granules of polymorphonuclear neutrophils and macrophages, which owns a potent bactericidal action that is mediated by production of hypochlorous acid from hydrogen peroxide and chloride ions [2, 3]. Generation of hypochlorous acid has been related to MPO and to this enzyme among the other animal hemoperoxidases [4]. MPO is also secreted in the extracellular space and increased plasma levels of MPO are promoted by inflammatory conditions in acute and chronic settings of cardiovascular patients [2]. A prognostic role of MPO has been reported in acute myocardial infarction, acute and chronic heart failure, and also healthy middle age or elderly subjects [5–8]. MPO contributes to the effects of oxidation and alterations of lipids and propagation of oxidative stress through chlorinating (halogenating) and peroxidase cycle activities [3]. MPO is also involved in the generation of nitrating species. In experimental and human HF, increased peroxynitrite (ONOO^−^) generation, which leads to extensive tyrosine protein nitration, derives from nitric oxide and superoxide or from MPO among the known animal hemoperoxidases [9, 10]. Tyrosine nitration along with cysteine oxidation may affect protein structure with a loss of function as we demonstrated in HF patients where Ceruloplasmin (Cp) showed a reduced FeO_*x*_I activity [11]. It has recently proposed that the physiological defense against the inappropriate action of MPO could be ascribed to Cp binding [12]. Cp, an alpha2-glycoprotein mainly synthesized by hepatocytes, whose functions include the transport of serum copper [13] and the acute phase inflammation reactant, is also involved in iron metabolism through its ferroxidase activity (FeO_*x*_) [14]. Cp is the main contributor of FeO_*x*_ activity in human plasma and is called FeO_*x*_I [15]. Cp has been suggested to be also a potent inhibitor of purified MPO, thus inhibiting production of hypochlorous acid even at low concentrations [16]. It has been demonstrated that, in plasma from Cp knock-out mice, MPO was able to act as a potent oxidizing enzyme, but no significant oxidation was observed in plasma from wild type animals where Cp was present [12, 16]. Cp and MPO binding has been suggested to be related to an electrostatic interaction between the cationic nature of MPO and the anionic charges of Cp [17]. It appears that Cp should provide a protective hedge against inadvertent oxidant production by MPO during inflammatory conditions (Figure 1). In the HF population, no data are available on the relationship between plasma MPO-related chlorinating activity and Cp and its FeO_*x*_I activity. Also even less known are the relationships between plasma chlorinating activity related to MPO and different parameters, expression of neurohormonal (BNP, norepinephrine, plasma renin activity, and aldosterone), inflammatory (high-sensitivity C-reactive protein (hs-CRP)), metabolic-nutritional (albumin and prealbumin), and oxidative (nitrated proteins, free malondialdehyde, and 15-F2t-isoprostane) domains. Based on these premises, we undertook a study on a cohort of stable chronic elderly HF of different severity compared to age-matched Controls, to investigate the above relationships and focusing in particular on the interaction of plasma MPO-related chlorinating activity with Cp-mediated FeO_*x*_I activity and with the other parameters linked to neurohormonal, inflammatory, nutritional, and oxidative/nitrosative domains.

## 2. Methods

### 2.1. Study Cohort and Follow-Up of Patients

Eighty-one consecutive stable chronic HF patients referred to the heart failure outpatient Clinic of the Cardiorenal Research Unit of the Department of Clinical and Experimental Medicine of the University Hospital of Parma were included in the present study. This group was a part of an original cohort of patients (81 of the 96 patients) already evaluated for nitrosative and oxidative stress in heart failure [11]. The diagnosis of HF was based on symptoms and clinical signs according to guidelines issued by the European Society of Cardiology [17] and by the American College of Cardiology [18]. The patients were free from clinical or laboratory signs of acute infection, rheumatoid or other autoimmune diseases, primary cachectic states (cancer, thyroid disease, severe liver disease, and severe chronic lung disease), neuromuscular disorders, myocardial infarction within the previous 20 weeks, diabetes mellitus, or severe chronic renal failure (serum creatinine level >2.0 mg/dL, >177 *μ*mol/L). Patients were clinically stable and on constant therapy at least 8 weeks prior to entering the study. The study was approved by the University of Parma Ethics Committee and complied with the Declaration of Helsinki, and all participants provided written informed consent.

Seventeen age-matched healthy subjects were recruited as Controls (CTR) from healthy subjects reporting for a periodical check-up at the cardiovascular prevention clinic of the same department. On study entry, a complete medical history, a physical examination, basal laboratory tests (serum creatinine, electrolytes, and lipid profile), plasma neurohormonal and inflammatory markers determination, an electrocardiogram, and an echocardiogram were obtained from all patients. Estimated glomerular filtration rate (eGFR) was calculated from the four-component Model of Disease in Renal Disease (MDRD) equation incorporating age, race, sex, and serum creatinine level: estimated eGFR = 186 *∗* (serum creatinine [in milligrams per deciliter])^−1.154^ *∗*  (age [in years])^−0.203^. For women, the product of the equation was multiplied by a correction factor of 0.742 [19].

### 2.2. Venous Blood Sampling Procedure and Biochemical Assays

Venous samples were collected as previously indicated [11, 20]. After at least 30 minutes of supine rest, blood was obtained from an indwelling catheter and collected in polypropylene tubes containing an EDTA (ethylenediamine tetraacetic acid) buffer (1.5 mg/mL), except for BNP where a mix of protease inhibitors (phenylmethylsulfonyl fluoride, trypsin inhibitor, and aprotinin 500 units/mL) was added. Except for FeO_*x*_I activity measurement, where fresh serum samples were used, multiple aliquots of plasma samples were stored at −80°C until assay time for norepinephrine, BNP, free malondialdehyde (MAD), total nitrated proteins, and Cp. All laboratory measurements were performed without any freeze-thaw cycles of the samples and by investigators blind to the clinical data.

Plasma chlorination activity, related to MPO, was measured in EDTA plasma samples by a colorimetric assay (OxiSelect Myeloperoxidase Chlorination Activity Assay Kit, Cell Biolabs, Inc., San Diego, CA, USA) evaluating hypochlorous acid generation by monitoring Cl-tau generation as previously described [21, 22]. Each sample from patients and Controls has been tested for 2 time points' determination (30 and 60 minutes of hydrogen peroxide incubation). Twenty-five *μ*L plasma sample from patients and Controls was mixed with 1 mM hydrogen peroxide solution according to the manufacturer instructions. After the generation of hypochlorous acid, the rapid reaction with taurine produced the stable taurine chloramine product. After adding a catalase-containing stop solution to block MPO catalysis by eliminating hydrogen peroxide, taurine chloramine reaction with TNB chromogen probe allowed measurement of MPO activity (absorbance at 405–412 nm). Data related to 60-minute incubation has been reported in the paper. The intra-assay and interassay coefficients of variation were 12% and 17% and the analytical sensitivity was 2.8 mU/mL. This assay measures plasma chlorinating activity that is related to MPO. Plasma samples from a subgroup of patients and CTR (one out of five patients) underwent MPO immunoprecipitation procedure to evaluate the contribution of MPO to plasma chlorinating activity. In the present study, chlorinating activity was found almost abolished in the supernatant after immunoprecipitation of MPO suggesting that chlorinating activity in plasma is mainly due to MPO (data not shown). A monoclonal anti-human anti-MPO antibody (Myeloperoxidase Antibody (1A1), Thermo Scientific Pierce Antibodies, Waltham, MA, USA) was cross-linked to Dynabeads protein G (Dynal Biotech, Oslo, Norway). Anti-MPO antibody was prepared from a stock solution of 1 mg/mL. After washing, 50 *μ*L of Dynabeads (1.5 mg) was resuspended after magnetic separation (Dynal MPC) in 0.1 M Na_2_HPO_4_ pH 8.0 and transferred to a polypropylene test tube. The solution was incubated with rotation (Dynal MX1-Mixer) for 20 minutes at room temperature with 200 *μ*L of phosphate buffer saline (pH 7.4) containing 6.5 *μ*g of antibody. After magnetic removal of supernatant, the beads-Ab complex was resuspended with phosphate buffer saline (pH 7.4) with 0.02% Tween 20. Two hundred and fifty *μ*L of diluted samples (1 : 25) from the patients and CTR was incubated with tilting and rotation for 60 minutes at room temperature. Test tubes were then placed on the magnet for 3 min to separate beads beads-Ab complex on the tube wall and the supernatant. Chlorinating activity was then measured in the supernatant.

FeO_*x*_I was measured by ferrous ion as substrate (Fe(II); ferrous ammonium) according to the method of Erel [23]. Norepinephrine, BNP, plasma renin activity, aldosterone, free MAD, high sensitivity C-reactive protein (hsCRP), Cp, albumin, and prealbumin were determined as previously described [11, 19]. Total nitrated proteins levels were assessed using a sandwich ELISA assay kit (Oxis Research International Inc., Foster City, CA USA). The intra-assay and interassay coefficients of variation were 4% and 14% and the analytical sensitivity was 2 nmol/L.

The test analytical sensitivity was 0.15 ng/mL for PRA, 7.6 pg/mL for aldosterone, and 3.0 pg/mL for BNP. hs-CRP was measured using the Dade Behring N Highly Sensitive CRP assay (Dade Behring Diagnostics) on the BN 100 Nephelometer. Plasma-free malondialdehyde, a marker of lipid peroxidation, was measured together with 15-F2t-isoprostane as oxidative pathway markers.

Plasma-free malondialdehyde was determined by HPLC-based thiobarbituric acid separation and spectrophotometric [11]. The intra-assay and interassay coefficients of variation were less than 10%. Plasma 15-F2t-ISO, after the extraction procedure, was measured by an enzyme immunoassay kit (Cayman Chemical, USA). Intra- and interassay coefficients of variation were 6 and 9%, respectively.

### 2.3. Data Analysis

Values are presented as mean ± SD or as median (range). Comparisons of the baseline characteristic variables among Controls and HF patients in NYHA Classes II, III, and IV were made with one-way analysis of variance or nonparametric equivalent Kruskal-Wallis one-way analysis of variance by ranks (depending on the parametric or nonparametric distribution) followed by Bonferroni* post hoc* or Dunn's test. Relations between parameters, including MPO-related chlorinating activity, FeO_*x*_I, Cp, nitrated protein, hsCRP, BNP, free MAD, albumin, prealbumin, and eGFR, were analyzed by linear regression analysis using Pearson or Spearman correlation coefficients. Lin-log plots are used to describe a semilog plot with a logarithmic scale on the *x*-axis and a linear scale on the *y*-axis or log-log plots to describe the relationship according to the distribution of the parameters. The D'Agostino-Pearson normality test was passed for all parameters, except for hsCRP, MPO-related chlorinating activity, and BNP that were log transformed to create a normal distribution. All statistical analyses were performed using SPSS for Windows 18.0 (SPSS Inc.). *P* < 0.05 was considered statistically significant.

## 3. Results

**Figure 2:**
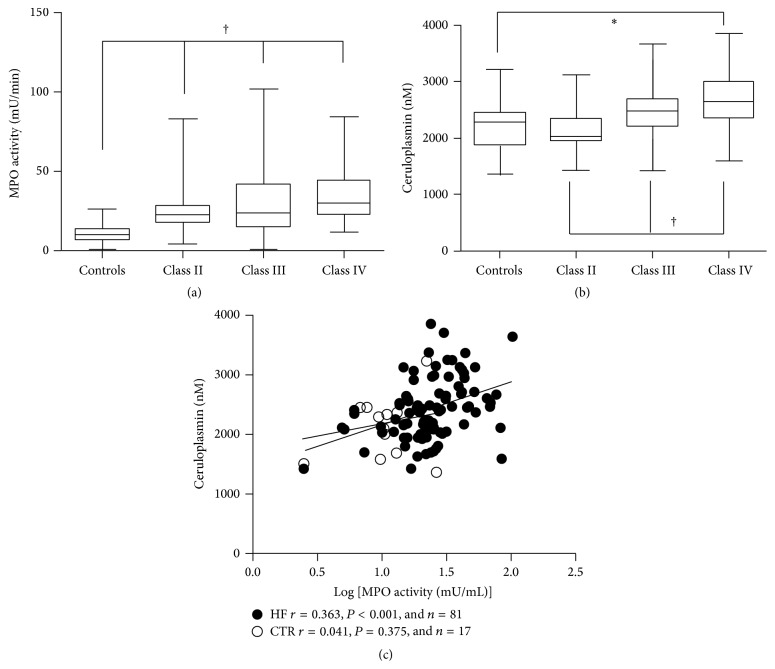
(a) Boxplots of serum MPO activity in Controls (*n* = 17) and heart failure patients (NYHA class II *n* = 26, III *n* = 29, and IV *n* = 26). One way ANOVA (*P* < 0.001) showed a significant difference among the groups (Classes II, II, and IV versus Controls, ^†^ *P* < 0.01). (b) Boxplots of serum Ceruloplasmin in Controls (*n* = 17) and heart failure patients (NYHA Classes II *n* = 26, III *n* = 29, and IV *n* = 26). One way ANOVA (*P* < 0.001) showed a significant difference among the groups (Classes IV and III versus Controls, ^†^ *P* < 0.01; Class IV versus Class II, ^*∗*^ *P* < 0.05). (c) Scatterplots of Myeloperoxidase chlorinating activity against Ceruloplasmin in HF patients and age-matched Controls. *r* = Spearman correlation coefficient.

Eighty-one HF patients were included and agreed to participate in the study (40 females and 41 males). Their mean age was 76 ± 9 years and their New York Heart Association (NYHA) functional class was separated in Class II/III/IV: 26/29/26, respectively. The clinical characteristics are indicated in Table 1 and clinical parameters were compared to age-matched CTR subjects (*n* = 17). Setting at 45%, the cut-off for EF, 52 (64%) HF patients had a reduced EF and 29 (36%) had a preserved EF. HF cause was ischemic in origin in about 81% of the patients, and 43% of them suffered from hypertension. Systolic and diastolic blood pressure were significantly lower in NYHA Class IV patients versus the other groups of patients (Table 1). Estimated GFR was reduced in the advanced HF Class (III and IV) compared to Controls and NYHA Class II patients. HF patients showed higher plasma levels of MPO-related chlorinating activity, Cp, BNP, norepinephrine, hsCRP, free MAD, nitrated protein, and 15-F2t-isoprostane as compared to CTR subjects, whereas FeO_*x*_I activity, albumin, and prealbumin were significantly reduced in HF versus CTR subjects (Table 2). A significant difference in MPO-related chlorinating activity was observed between HF patients and CTR, with an incremental trend from NYHA II to NYHA class IV (Figure 2(a) and Table 2). No differences were observed in MPO-related chlorinating activity between HF patients with reduced or preserved EF. Cp levels were higher in NYHA Classes III (+16%) and IV (+24%) as compared to NYHA Class II (*P* < 0.05) (Figure 2(b)). FeO_*x*_I activity was reduced in Class IV HF patients compared to NYHA Class II patients (−23%) and Controls (−24%) as indicated in Table 2.

**Figure 3:**
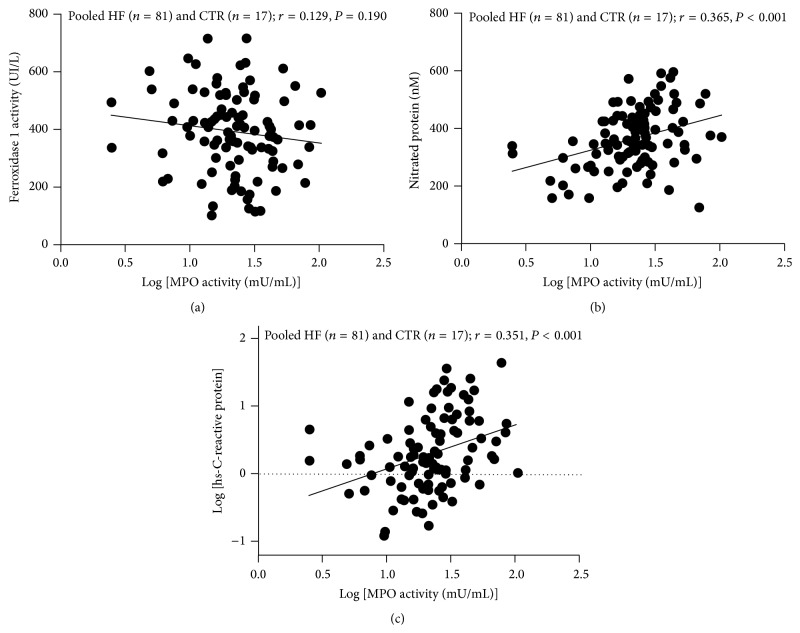
Scatterplots of Myeloperoxidase chlorinating activity against Ferroxidase I Activity (a), nitrated protein (b), and high sensitivity C-reactive protein (c) in pooled subjects patients (pooled HF patients (*n* = 81) and age-matched Controls (*n* = 17)). *r* = Spearman correlation coefficient.

**Figure 4:**
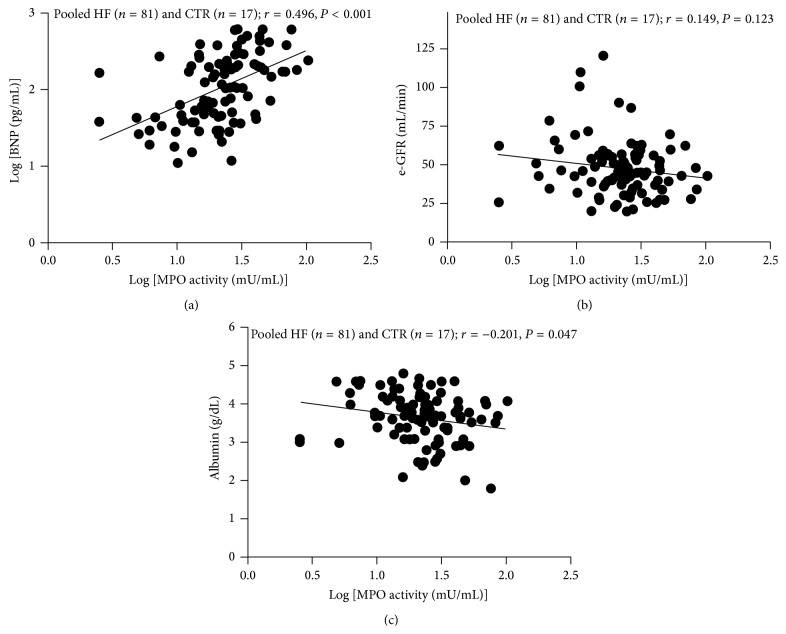
Scatterplots of Myeloperoxidase chlorinating activity against BNP (a), eGFR estimated glomerular filtration rate (b), and albumin (c) in pooled subjects patients (pooled HF patients (*n* = 81) and age-matched Controls (*n* = 17)). *r* = Spearman correlation coefficient.

In HF patients, a close correlation was found between plasma MPO-related chlorinating activity and CP levels (*r* = 0.363, *P* < 0.001, and *n* = 81) whereas no correlation was found between plasma MPO chlorinating activity and FeO_*x*_I activity (*r* = 0.129 and *P* = 0.190, Figure 3(a)). A positive linear relationship was observed between MPO-related chlorinating activity and nitrated protein (*r* = 0.365 and *P* < 0.001, Figure 3(b)), hsCRP (*r* = 0.351 and *P* < 0.001, Figure 3(c)). The strongest positive relationship was found between chlorinating activity and BNP (*r* = 0.496 and *P* < 0.001, Figure 4(a)), and no correlation was observed between MPO-related chlorinating activity and eGRF (*r* = 0.149 and *P* = 0.123, Figure 4(b)). A borderline negative correlation was found between MPO-related chlorinating activity and albumin (*r* = −0.201 and *P* = 0.047, Figure 4(c)).

## 4. Discussion

There are several results arising from this study on a cohort of chronic HF patients with both reduced and preserved EF. First plasma MPO-related chlorinating activity is elevated in elderly HF patients, with increasing levels linked to the worsening of NYHA class, compared with age-matched Controls. We measured plasma MPO-related chlorinating activity and not MPO mass and we observed that no differences were evident between reduced and preserved EF HF patients. Second, we reported a positive correlation between plasma MPO-related chlorinating activity and Cp levels in HF patients. This finding in part contrasts with what was expected. Cp binding to MPO should represent a protective shield against increased oxidant production by MPO, also in HF patients. Third, plasma MPO-related chlorinating activity is positively associated with several systemic inflammatory, neurohormonal, and oxidative/nitrosative parameters expressing the activation of these pathways in HF patients while progressing the disease. Fourth, a negative relationship has been found between with the MPO-related chlorinating activity and nutritional parameters. All these findings deserve specific comments.

First, we confirm what is already known that MPO-related chlorinating activity in HF patients is increased even if we do not have information on MPO enzyme mass levels. Circulating MPO enzyme levels are largely derived from the secretion of this enzyme from leukocytes in the blood stream after inflammatory activation. The process that leads to hypochlorous acid from hydrogen peroxide and chloride ions was always thought to be a unique characteristic of MPO excluding from this the other mammalian hemoperoxidases (eosinophil peroxidase, lactoperoxidase, and thyroid peroxidase) [4]. However, in a recent study, Li et al. identified the vascular peroxidase 1 as a new member of the family of heme peroxidase capable of producing small amounts of hypochlorous acid starting from chloride and hydrogen peroxide [22]. The majority of commercially available assays do not directly measure MPO enzymatic activity in plasma but the amount of the enzyme mass by enzyme-linked immunosorbent or chemiluminescent automated assay [24]. In the present study, we report the MPO-related chlorination activity of plasma from HF patients and found its activity increased while the severity of HF progresses.

We also investigated the relationship between MPO-related chlorinating activity and Cp levels and found a close positive association (Spearman's *r* 0.363, *P* < 0.001). As recently shown, Cp is considered a strong inhibitor of MPO activity, with a marked reduction of chlorination activity even at low concentration [12]. In our study, we were expecting a possible inverse association between MPO chlorinating activity and Cp circulating levels but the opposite was observed in HF patients. In addition, no correlation was found between Cp-related FeO_*x*_I activity and MPO-related chlorinating activity. It has recently been reported in literature that Cp levels are increased while increasing the severity of HF and probably reflecting the inflammatory status of these patients. Some evidences have also shown a strong independent prognostic value of high Cp circulating levels in stable patients undergoing elective coronarography and in a group of patients without HF or cardiovascular disease taken from the Atherosclerosis Risk in Communities Study [25, 26]. In our recent study, Cp circulating levels were not able to predict mortality, while it was Cp-related FeO_*x*_I activity [11].

In the present paper, we showed the increased levels of circulating nitrated proteins in HF patients compared to Controls. A close positive association has been found between MPO-related chlorinating activity and circulating nitrated proteins. Our results agree with other studies reporting that severely diseased HF patients express the highest levels of plasma nitrated proteins [11, 27]. Protein nitrotyrosine formation has been claimed as a “footprint” for ONOO^−^ generation [1, 28–31] but recently alternative mechanisms of nitration have been shown to take place* in vivo*, involving the generation of the NO_2_^∙^ radical by MPO and also eosinophil peroxidase [10, 13, 27, 30].

In our study, we reported a strong association between MPO-related chlorinating activity and hsCRP. This finding underlines the participation of a systemic inflammatory process in HF progression. Such observation agrees with a series of studies in different cohorts of patients where the associations between MPO and inflammation in acute and chronic setting of coronary heart disease and in chronic systolic HF patients as well in other populations of patients such as hemodialysis patients were demonstrated [32–35].

However, in a recent and well-performed study in chronic systolic heart failure patients, Wilson Tang et al. did not observe the association between MPO (measured as mass and not chlorinating activity) and hsCRP [36]. The lack of association was somewhat unexpected and the authors suggested that MPO levels allow the differentiation of the leukocyte-based pathophysiologic contribution to cardiovascular disease from a generalized systemic inflammatory process that was more mirrored with hsCRP [36]. Some differences were detectable in their cohort of patients from the patients included in our study: their patients were younger (mean 57 years) and had systolic HF whereas in our group also preserved HF patients were included and they have better renal function.

In our study, we also showed a direct relationship between chlorinating activity and neurohormonal activation parameters, in particular BNP and norepinephrine. The closest association was with BNP in a Spearman coefficient *r* close to 0.5. In our study, renal function does not correlate with plasma MPO-related chlorinating activity in patients with HF: patients in NYHA Classes III and IV showed a reduction of 25–30% of the eGFR compared to Controls.

An interesting finding of our study is the observation of reduced levels of albumin and prealbumin in the advanced HF patients (Class NYHA IV versus the other Class and Controls) suggesting a poorer nutritional status. It has never been reported before in HF patients of an inverse relationship between MPO-related chlorinating activity and circulating levels of albumin. Protein malnutrition is a phenomenon that could be observed in HF when patients develop a state of cachexia and represents a serious negative prognostic factor. Both albumin and prealbumin values could be lowered while aging. In addition, albumin that can reflect the nutritional status can also be influenced by the chronic low inflammatory status accompanying the time course of HF disease.

The present study did not investigate the prognostic role of MPO-related chlorinating activity, which has already been suggested in various clinical cardiovascular conditions to identify patients at increased risk for progressive cardiac deterioration [32–36], but we explore the association with known mechanisms of progression of disease severity. Our cross-sectional study limits the interpretation of our findings. Although the association between increasing plasma MPO-related chlorinating activity and increasing HF severity does not prove a cause-and-effect relation, thinking of chlorinating activity as a disease marker without pathophysiological properties is reductive and it is still intriguing to note that MPO chlorinating activity appears to be involved in the increased nitration observed in HF patients and therefore an active contributor to disease progression. In conclusion, our findings provide insight into the interaction between MPO-related chlorinating activity, Cp, and other biomarkers, expressing different domains such as neurohormonal, inflammatory, metabolic-nutritional, and oxidative domains, all potentially involved in the prognosis of HF patients.

## Figures and Tables

**Table 1:** Clinical characteristics of heart failure patients and healthy Controls.

	Controls (*n* = 17)	NYHA Class II (*n* = 26)	NYHA Class III (*n* = 29)	NYHA Class IV (*n* = 26)
Age, years	76 ± 11	76 ± 7	77 ± 10	75 ± 9
Gender, male	7	13	9	20
BMI (kg/m^2^)	23.7 ± 4.0	25.7 ± 2.9	23.8 ± 3.7	23.5 ± 3.6
Systolic BP (mm Hg)	135 ± 23	138 ± 19	136 ± 17	113 ± 19^*∗*†#^
Diastolic BP (mm Hg)	73 ± 8	80 ± 14	79 ± 12	64 ± 12^†#^
Heart rate (bpm)	81 ± 13	78 ± 11	72 ± 11	80 ± 11
Ischemia/hypertense/idiopathic	—	20/13/0	24/14/0	22/6/2
Current smoker (%)	47	35	14	35
Ejection fraction (%)	66 ± 6	51 ± 6^*∗*^	41 ± 7^*∗*†^	29 ± 7^*∗*†#^
Haemoglobin (g/dL)	13.2 ± 1.0	12.9 ± 0.9	13.0 ± 1.4	12.6 ± 1.1
Neutrophils (10^3^ cell/*μ*L)	2.70 ± 0.92	3.73 ± 1.43^*∗*^	3.51 ± 1.27^*∗*^	3.66 ± 1.21^*∗*^
Sodium (mEq/L)	141 ± 4	141 ± 4	138 ± 3	135 ± 5^*∗*†^
eGFR (mL/min)	60 ± 22	49 ± 15	45 ± 18^*∗*^	41 ± 14^*∗*^

Data are reported as mean ± SD; eGFR: estimated glomerular filtration rate; *∗* indicates *P* less than 0.05 versus Controls, † versus NYHA II, and # versus NYHA III.

**Table 2:** Oxidative, neurohormonal, inflammatory, and nutritional parameters of heart failure patients and healthy Controls.

	Controls (*n* = 17)	NYHA Class II (*n* = 26)	NYHA Class III (*n* = 29)	NYHA Class IV (*n* = 26)
Oxidative				
MPO activity (mU/min)	10.5 (2.5–26.4)	21.9 (4.8–83.1)^*∗*^	23.5 (2.5–102.5)^*∗*^	30.1 (12.2–85.2)^*∗*^
Ceruloplasmin (nmol/L)	2176 ± 453	2153 ± 426	2508 ± 489^†^	2662 ± 560^*∗*†^
FeO_*x*_ I activity (UI/L)	442 ± 128	437 ± 142	367 ± 151	336 ± 110^*∗*†^
Nitrated proteins (nmol/L)	274 ± 69	314 ± 75	402 ± 97^*∗*†^	428 ± 85^*∗*†^
Malondialdehyde (umol/L)	0.25 ± 0.09	0.32 ± 0.09	0.43 ± 0.13^*∗*†^	0.47 ± 0.12^*∗*†^
15-F2t-isoprostane, pg/mL	56 ± 30	91 ± 30	128 ± 48^*∗*†^	140 ± 46^*∗*†^
Neurohormonal				
Norepinephrine (pg/mL)	256 ± 76	266 ± 70	363 ± 101^*∗*†^	621 ± 220^*∗*†#^
BNP (pg/mL)	37 (11–62)	48 (12–196)	183 (19–459)^*∗*†^	283 (105–620)^*∗*†^
PRA, ng/mL/hr	1.12 ± 0.86	1.58 ± 0.81	2.41 ± 1.24^*∗*^	4.69 ± 2.22^*∗*†#^
Aldosterone, pg/mL	169 ± 79	177 ± 94	247 ± 135	295 ± 110^*∗*†^
Inflammatory and nutritional				
hsCRP (mg/dL)	0.78 (0.12–4.56)	0.82 (0.17–9.30)	1.90 (0.66–36.16)^*∗*†^	7.22 (1.49–44.31)^*∗*†#^
Albumin (g/dL)	3.9 ± 0.5	3.9 ± 0.5	3.7 ± 0.6	3.1 ± 0.7^*∗*†#^
Prealbumin, mg/dL	29.5 ± 5.3	29.8 ± 6.3	26.5 ± 7.7	20.2 ± 8.1^*∗*†^
Total cholesterol (mg/dL)	194 ± 20	216 ± 34	210 ± 42	202 ± 36

Data are reported as mean ± SD or median (range) depending on the distribution of data; BNP: B type natriuretic peptide; hsCRP: high sensitivity C-reactive protein; PRA: plasma renin activity; MPO: Myeloperoxidase-related chlorinating activity; FeO_*x*_ I: ferroxidase I activity; *∗* indicates *P* less than 0.05 versus Controls, † versus NYHA II, and # versus NYHA III.
